# Oxytocin-induced antinociception in the spinal cord is mediated by a subpopulation of glutamatergic neurons in lamina I-II which amplify GABAergic inhibition

**DOI:** 10.1186/1744-8069-4-19

**Published:** 2008-05-29

**Authors:** Jean-Didier Breton, Pierre Veinante, Sandra Uhl-Bronner, Angela Maria Vergnano, Marie José Freund-Mercier, Rémy Schlichter, Pierrick Poisbeau

**Affiliations:** 1 Department Nociception and Pain, Institut des Neurosciences Cellulaires et Intégratives, Unité Mixte de Recherche 7168, Centre National de la Recherche Scientifique/Université Louis Pasteur, Strasbourg, France

## Abstract

**Background:**

Recent evidence suggests that oxytocin (OT), secreted in the superficial spinal cord dorsal horn by descending axons of paraventricular hypothalamic nucleus (PVN) neurons, produces antinociception and analgesia. The spinal mechanism of OT is, however, still unclear and requires further investigation. We have used patch clamp recording of lamina II neurons in spinal cord slices and immunocytochemistry in order to identify PVN-activated neurons in the superficial layers of the spinal cord and attempted to determine how this neuronal population may lead to OT-mediated antinociception.

**Results:**

We show that OT released during PVN stimulation specifically activates a subpopulation of lamina II glutamatergic interneurons which are localized in the most superficial layers of the dorsal horn of the spinal cord (lamina I-II). This OT-specific stimulation of glutamatergic neurons allows the recruitment of all GABAergic interneurons in lamina II which produces a generalized elevation of local inhibition, a phenomenon which might explain the reduction of incoming Aδ and C primary afferent-mediated sensory messages.

**Conclusion:**

Our results obtained in lamina II of the spinal cord provide the first clear evidence of a specific local neuronal network that is activated by OT release to induce antinociception. This OT-specific pathway might represent a novel and interesting therapeutic target for the management of neuropathic and inflammatory pain.

## Background

Oxytocin (OT) is a nonapeptide synthesized in magnocellular neurons of the paraventricular and supraoptic nuclei of the hypothalamus and acts as a neurohormone during parturition and milk ejection reflex [1]. It is also synthesized in parvocellular neurons of the paraventricular nucleus (PVN) which project to various areas of the central nervous system including the spinal cord. In the spinal cord, oxytocinergic projection sites [2-5] match well OT binding sites [6,7] in the superficial layers of the dorsal horn and in the autonomic regions (intermediolateral columns, intermediomedial grey matter, lamina X and sacral parasympathetic nucleus). Using electron microscopy, OT-positive synaptic terminals were found to form mainly axodendritic synapses on lamina I-II neurons [8].

Although some conflicting results have been reported in the literature [9,10], analgesic effects of OT have been reported in most studies after systemic and/or central administration of OT in naive rodents [11] or during the development of inflammatory [12-14] or neuropathic pain [15]. An OT-sensitive antinociception can also be induced in rats by massage-like stimulation [16,17], swim stress [18] and electrical stimulation the PVN in naïve [19,20] and neuropathic rats [21,22], suggesting the involvement of an endogenous OT receptor-dependent analgesic system.

Little is known about the possible mechanisms and neuronal networks mediating these antinociceptive effects of OT in the spinal cord. PVN stimulation or OT infusion reduced the incoming peripheral Aδ and C-fiber activation in dorsal horn spinal neurons [21,23]. It should be noted here that most of these in vivo results were obtained from neurons receiving convergent sensory informations (from Aβ, Aδ and C fibers) and recorded in deep layers (Lamina III-V) of the dorsal horn. In some rare cases, neurons were also shown to be excited after application of OT [23] or after PVN stimulation [24]. However, in spinal cord slices of rats and mice, OT reduced electrically-evoked glutamatergic synaptic currents between primary afferents and lamina II neurons [18] whereas in primary cultures of neonatal laminae I-III dorsal horn neurons, OT produced a facilitation of glutamatergic synaptic transmission via a presynaptic mechanism of action [25]. Finally, OT was shown to inhibit glutamate-induced excitation of spinal neurons in vivo [23], and, in a recent study, antinociceptive action of spinal OT is proposed to be mediated by a GABAergic mechanism [24].

In order to clarify the effects of OT in spinal antinociception, we determined its effects on excitatory and inhibitory synaptic transmission in lamina II, a layer containing mainly local interneurons which are thought to play an important role in spinal pain processing by controlling the local balance between excitation and inhibition. Using electrophysiological and morphological tools, we characterized the effects of OT on lamina II neurons, identified a subpopulation of OT-sensitive postsynaptic target neurons, specify their neurochemical identity and their role in recruiting a local interneuronal network leading to antinociception.

## Results

### Characterization of fast spontaneous synaptic transmission in lamina II interneurons

**Figure 1:**
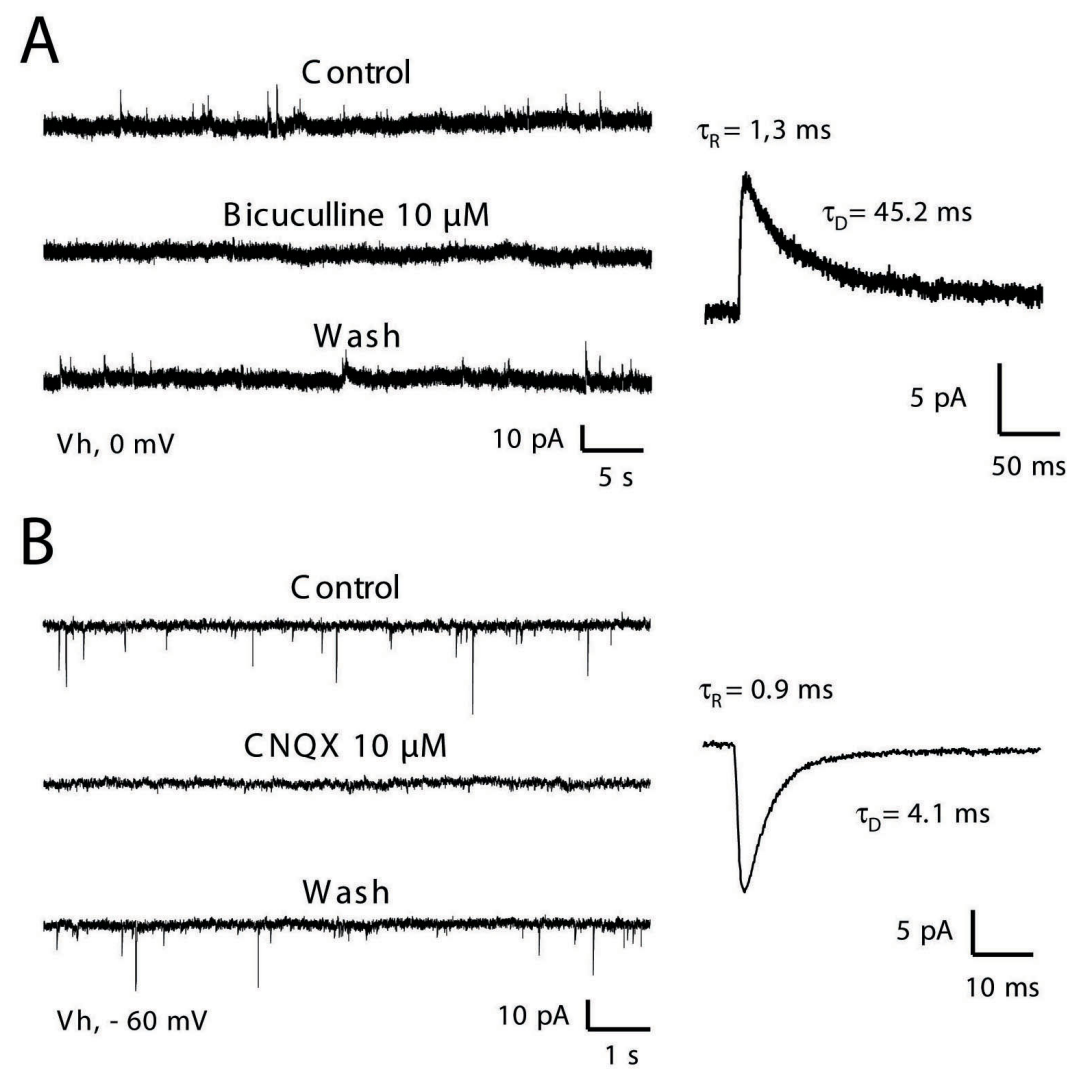
**characterization of fast spontaneous inhibitory and excitatory synaptic currents in lamina II**. Glycine-receptor-mediated sIPSCs were blocked in these experiments by adding 1 μM strychnine in the general bath perfusion. **A**: GABA_A_-receptor-mediated sIPSCs (GABAA-R sIPSCs) were identified as outward currents (left panel) at a holding potential of 0 mV. These sIPSCs were reversibly blocked in the presence of 10 μM bicuculline, a selective antagonist of GABAA-Rs. Panel on the right illustrates an averaged current obtained from 46 individual traces. GABAA-R sIPSCs were best described by a monoexponential decay time constant in both the activation phase (τ_R _= 1.3 ms) and deactivation phase (τ_D _= 45.2 ms). **B**: At a holding potential of -60 mV (i.e. at the equilibrium potential for Cl^- ^ions), only fast inward currents were detected (left panel). These events were AMPA-type glutamate-receptor-mediated sEPSCs (AMPA-R sEPSCs) since they were reversibly inhibited in the presence of CNQX (10 μM) a specific blocker of AMPA subtype glutamate receptor. The trace on the right was obtained by averaging 168 individual AMPA-R sEPSCs. These sEPSCs were characterized by a τ_R _of 0.9 ms and τ_D _of 4.1 ms).

In all lamina II neurons recorded in the whole-cell configuration of the patch-clamp technique (n = 39), spontaneous inhibitory and excitatory postsynaptic currents (sIPSCs and sEPSCs, respectively) were observed (Figure 1). Under our experimental conditions, the chloride equilibrium potential was set at -60 mV, and IPSCs were recorded as transient outward currents when lamina II neurons were held at 0 mV (Figure 1A). Both glycine- and GABA_A _receptor-mediated sIPSCs (GABAA-R sIPSCs) were present and were blocked by 1 μM strychnine (not shown) and 10 μM bicuculline respectively (Figure 1A). We found that pharmacologically-isolated GABAA-R sIPSCs occurred at a mean frequency of 0.12 ± 0.02 Hz and exhibited a mean amplitude of 11.1 ± 1.5 pA (n = 6). For comparison, fast glutamatergic sEPSCs were detected as transient inward currents at a holding potential of -60 mV (Figure 1B). They occurred at a mean frequency of 1.26 ± 0.47 Hz and had a mean amplitude of -16.3 ± 0.8 pA (n = 16). These currents were blocked by 6-cyano-7-nitroquinoxaline-2,3-dione (CNQX, 10 μM) (Figure 1B), indicating that they were mediated by the AMPA subtype of glutamate receptors. The detailed properties of the kinetics of each type of current are given in Table 1 for spontaneous and miniature (i.e. recorded in the presence of 0.5 μM of TTX) synaptic events.

**Table 1:** Characteristics of synaptic events mediated by GABA_A _and AMPA receptors.

	Amplitude (pA)	τ_R _(ms)	τ_D _(ms)	Frequency (Hz)	n
GABAA-R mIPSCs	11.7 ± 0.8	3.17 ± 0.55	29.06 ± 3.83	0.13 ± 0.03	6
GABAA-R sIPSCs	11.1 ± 1.5	3.97 ± 0.72	30.35 ± 0.86	0.12 ± 0.02	6
AMPA-R mEPSCs	-14.1 ± 0.9	1.27 ± 0.07	3.91 ± 0.17	0.81 ± 0.17	14
AMPA-R sEPSCs	-16.3 ± 0.8	1.31 ± 0.10	4.23 ± 0.26	1.26 ± 0.47	16

Mean values and SEM are shown for amplitude, rise and decaying monoexponential time constants (τ_R _and τ_D_, respectively) and frequency of occurrence of spontaneous (sIPSCs and sEPSCs) and miniature (mIPSCs and mEPSCs) events. Note that the mean values were not statistically different while recorded in the absence (spontaneous) or in the presence of tetrodotoxin (miniatures).

### Consequences of oxytocin agonist application (TGOT) on spontaneous synaptic transmission recorded in lamina II neurons

**Figure 2:**
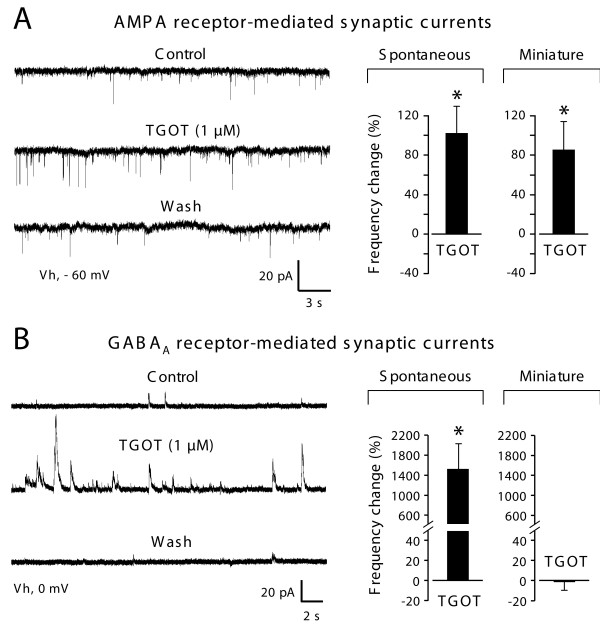
**Differential effects of a selective oxytocin receptor (TGOT) on glutamatergic and GABAergic synaptic currents**. **A**: Raw recordings of spontaneous AMPA-R sEPSCs (left traces) illustrate the increase of frequency triggered by the application of TGOT (1 μM). This effect was reversible and observed in 35.7% of the lamina II neurons. Histograms on the right show the mean increase (± SEM) in frequency (with respect to control) of sEPSCs and mEPSCs. **B**: Elevation in the frequency of occurrence of GABAA-R sIPSCs was observed in all lamina II neurons recorded (n = 6 out of 6; raw traces and histogram on the left). However, TGOT never changed the frequency of miniature GABAA-R events (right histogram). Asterisks indicate statistical significance using Student's t-test, p < 0.05.

Spontaneous AMPA-receptor-mediated EPSCs (AMPA-R sEPSCs) were recorded in the presence of 1 μM strychnine and 10 μM bicuculline (Figure 2A). Bath application of the selective oxytocin receptor agonist (TGOT 1 μM) increased the frequency of occurrence (but not the amplitude) of spontaneous AMPA-R sEPSCs (Figure 2A, histogram) in 5 out of 14 lamina II neurons (35.7%). This frequency of sEPSCs was of 1.04 ± 0.38 Hz under control conditions and increased to 1.79 ± 0.55 Hz in the presence of TGOT (n = 5). This change was reversible and found to be statistically significant (p < 0.05) and represented an average increase of 103 ± 27%. By contrast to its relatively modest effect on sEPSCs, TGOT (1 μM) induced a significant increase in the amplitude (Control: 14.4 ± 1.8 pA; TGOT: 25.5 ± 2.6 pA, n = 6; p < 0.05) and frequency of GABAA-R sIPSCs (Control: 0.12 ± 0.02 Hz; TGOT: 1.69 ± 0.61 Hz, n = 6; p < 0.05) in all lamina II neurons from which we recorded (n = 6 out of 6). The effect of TGOT was fully reversible after 10–15 minutes of washout and was never observed in the presence of OTA (d(CH_2_)^5 ^- [Tyr(Me)^2^, Thr^4^-Tyr-NH_2_^9^] ornithine vasotocin, 1 μM), a selective OT receptor antagonists (n = 5, not shown). This result indicated that the increase in frequency of sIPSCs was mediated by the activation of OT receptors.

### Presynaptic OT receptors on glutamatergic synaptic terminals

In the presence of TTX (0.5 μM), TGOT also stimulated the frequency of AMPA-R mEPSCs (Figure 2A, left panel and histogram) in a subpopulation (50%, 6 out of 12) of lamina II neurons. The mean frequency of mEPSCs increased from 1.27 ± 0.30 Hz under control conditions to 2.31 ± 0.65 Hz in the presence of TGOT (1 μM). This change in frequency was statistically significant (p < 0.05) and represented an average increase of +96.8 ± 31.0% (n = 6; Figure 2A, right histogram) which was similar to that observed for sEPSCs. In sharp contrast with this result, TGOT failed to change the frequency of occurrence of GABAA-R mIPSCs (Figure 2B, right histogram) since, in all lamina II neurons recorded, the mean frequency remained stable (Control: 0.13 ± 0.03 Hz; TGOT: 0.12 ± 0.04 Hz, n = 6; p > 0.05; average change: -1.2 ± 9.1%, n = 6). TGOT had no apparent effect on the frequency of glycine receptor-mediated spontaneous and miniature synaptic transmission (not shown).

### TGOT reveals a novel population of AMPA-R mEPSCs having slow rise and decay kinetics

**Figure 3:**
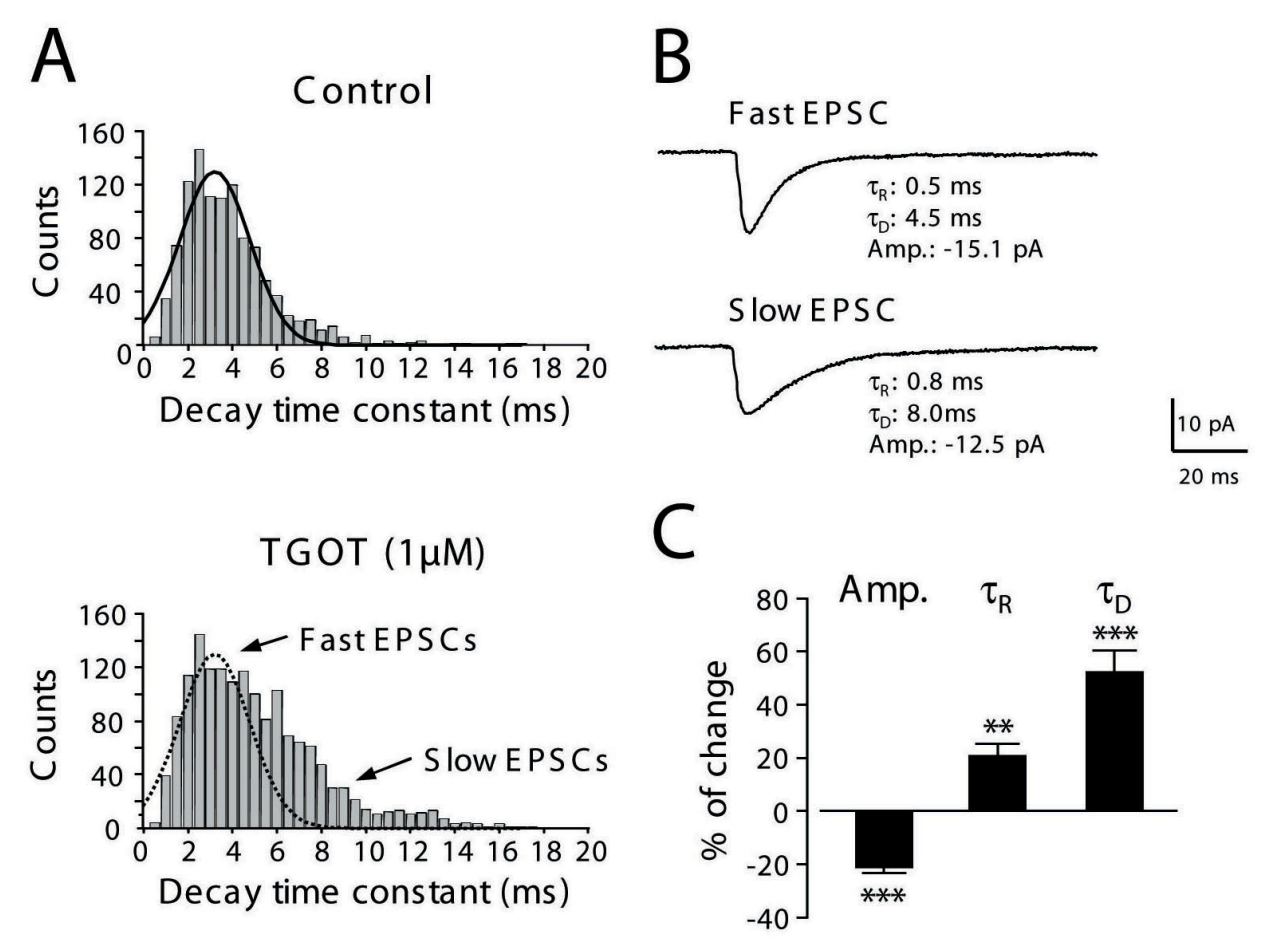
**Application of TGOT reveals a population of AMPA-R miniature EPSCs with slow activation and deactivation kinetics**. **A**: The histograms show the distribution of the decay time constants (τ_D_) of miniature AMPA-R EPSCs pooled from 6 cells and detected during an identical time period of 5 minutes before (top, n = 1075 events) and after the application of 1 μM TGOT (bottom, n = 1563 events). In the presence of TGOT, the appearance of slowly decaying mEPSCs was noted in addition to the fast mEPSC population of mEPSCs with faster kinetics also found in the control situation. **B**: Averaged currents obtained from 91 individual mEPSCs of representative recorded neurons during TGOT treatment. Compared to the fast mEPSCs (upper trace), the averages of slow mEPSCs obtained in the presence of TGOT (lower trace) exhibited longer monoexponential constants for rise time (from τ_R _= 0.5 ms in control to τ_R _= 0.8 ms in TGOT) and decaying phases (from τ_D _= 4.5 ms in control to τ_D_= 8.0 ms in OT). The mean amplitude was also reduced in the presence of TGOT. **C**: Graph summarizing the changes observed in the mean amplitude (Amp.), rise time (τ_R_), and decay time constants (τ_D_) of AMPA-R mEPSCs during perfusion of TGOT. TGOT-induced increase in frequency was always associated with the appearance of slow rising and decaying currents having lower amplitudes. All changes observed during TGOT application were significant: p < 0.01 (**: τ_R_, n = 6), and p < 0.001 (***: Amp and τ_D_, n = 6) using the Student's t-test.

Detailed analysis of mEPSC kinetics revealed that in the presence of TGOT a subpopulation of mEPSCs remained unchanged whereas an additional population of mEPSCs seemed to appear. As illustrated in Figure 3B–C, this "novel" population of miniature AMPA-R EPSCs had larger time to peak values (τ_R_: +20.9 ± 4.6%, n = 6, p < 0.01), slower deactivation time constants (τ_D_: +52.7 ± 7.9%; n = 6, p < 0.001) and smaller mean amplitudes (Amp.: -20.9 ± 2.0%; n = 6, p < 0.001). The effect of TGOT became particularly clear when the distribution of the monoexponential decay time constants of AMPA-R mEPSCs was represented under the form of a binned histogram (Figure 3A). Under control conditions (Figure 3A, top histogram), the distribution of the decay time constants could be adjusted by a Gaussian distribution centered at 3.21 ms, while in the presence of TGOT this distribution was strongly asymmetric because of the presence of the "newly" detected mEPSCs with slow decay time constants (Figure 3A, bottom histogram). These TGOT-sensitive mEPSCs were fully blocked by CNQX (10 μM) and disappeared upon washout of TGOT.

These results suggest that presynaptic OT receptors facilitate glutamatergic synaptic transmission by revealing a novel or previously silent or undetected population of EPSCs.

### Immunohistochemical identification of PVN-activated (OT-sensitive) dorsal horn neurons

**Figure 4:**
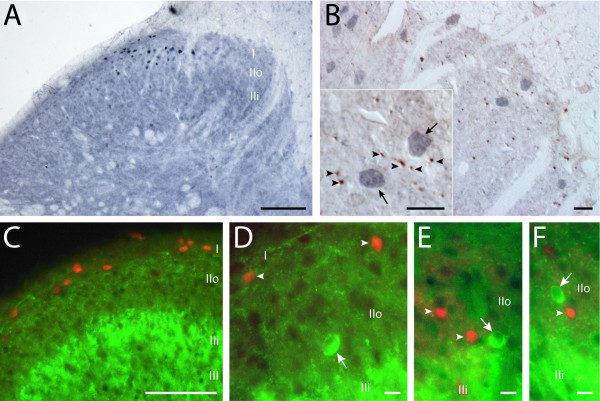
**c-Fos expressing cells in the superficial layers of the dorsal horn after electrical stimulation of the ipsilateral PVN**. **A: **Labeled c-Fos positive nuclei were localized in the most superficial layers i.e. lamina I and outer lamina II (IIo). **B**: Double immunoperoxidase labeling with an antibody against NpOT showed NpOT positive fibers (brown; arrowheads on inset) in the vicinity of c-Fos expressing nuclei (blue grey; arrows on inset). **C: **Double immunofluorescence labeling with an antibody against glutamic acid-decarboxylase (GAD) showed c-Fos positive nuclei (red cy3 fluorescence) in lamina I and IIo exhibiting a low GAD-immunoreactivity (green FITC fluorescence) whereas lamina IIi was strongly labeled by GAD antibody. **D, E, F**: In double immunofluorescence, c-Fos positive nuclei (arrowheads) never exhibited GAD labeled cytoplasm, despite the proximity in lamina IIo of GAD-immunoreactive neurons (arrows). A: 25 μm thick section; B: 1.5 μm semi-thin section; C-F: 30 μm thick sections. Sale bars: 100 μm in A and C; 10 μm in B and D-F. Individual colour channels on merged images were adjusted in C-F.

**Figure 5:**
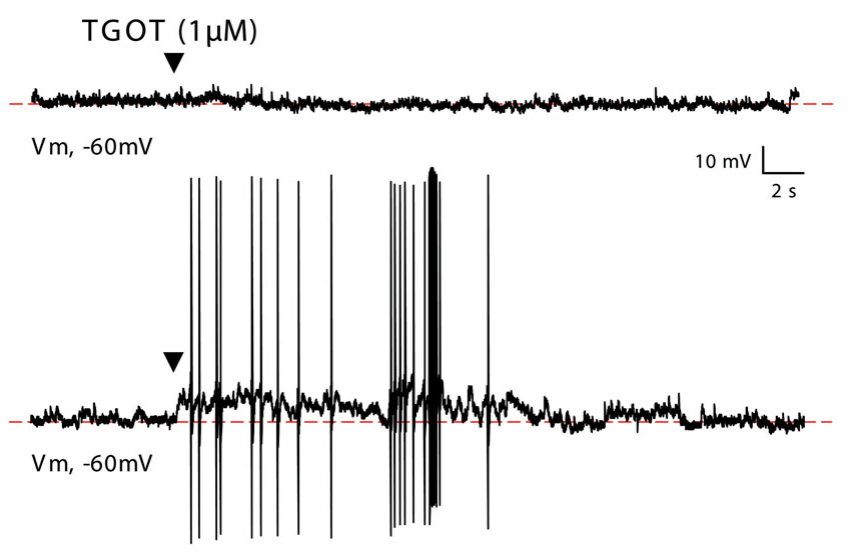
**Effect of TGOT on the membrane potential (Em) of lamina II neurons**. Raw traces illustrating the effect of TGOT (1 μM, black arrowhead) on two different lamina II neurons. In the vast majority (90%) of lamina II neurons (top trace), TGOT application had no apparent effect on Em. In a small number of neurons (10%), TGOT induced a transient depolarization which reached the threshold value for the emission of action potentials (bottom trace).

In order to localize and to tentatively identify the phenotype of the lamina II neurons activated upon OT release, we used an immunohistochemical approach based on c-Fos nuclear expression following PVN electrical stimulation in anaesthetized rats. As shown in Figure 4A and 4C, the c-Fos positive nuclei of PVN-activated cells in the spinal cord were rare and systematically localized in the most superficial layers of the dorsal horn (lamina I and outer part of lamina II: I/IIo) as well as in the sympathetic intermedio-lateral column (not shown). Lamina I/IIo cells immunopositive for c-Fos were mainly found in the ipsilateral dorsal horn, with a slight preference for the medial half of superficial laminae versus lateral half. This labeling matched the distribution of OT-positive fibers and OT-binding sites (see additional file 1). In good agreement, OT-containing fibers (brown staining in Figure 4B, arrowheads) could be found close to the c-Fos positive nuclei (blue staining in Figure 4B, arrows). PVN-activated cells were identified as neurons because c-Fos-positive nuclei were always surrounded by a thin (perinuclear) cytoplasmic compartment which was immunopositive for the neuronal marker MAP2 and negative for the astroglial marker GFAP (not shown). In a double immunofluorescence protocol against c-Fos and glutamic acid decarboxylase (GAD), the enzyme synthesizing GABA, c-Fos positive nuclei of PVN-activated cells were found in laminae with poor GAD-immunoreactivity (I/IIo) while lamina IIi showed a strong GAD-immunoreactivity (Figure 4C). Among 720 c-Fos positive nuclei observed in lumbar segments of two animals (80% in lamina I, 20% in lamina IIo), none were found in GAD positive neurons (Figure 4D–F), thus suggesting that PVN-activated neurons represented a population of non-GABAergic, i.e. glutamatergic neurons. In addition, we performed patch-clamp recordings of lamina II neurons in the current clamp mode to characterize the effect of TGOT application on the membrane potential at rest (i.e. no current injection: i = 0). As illustrated in figure 5 (top trace), TGOT superfusion (1 μM) did not modify the membrane potential (control: -63.6 ± 1.7 mV; TGOT: -60.8 ± 2.0 mV; n = 19; p > 0.05) in the vast majority of the recorded lamina II neurons (n = 19 neurons out of 21; 90.5%). Only a small fraction of neurons were depolarized transiently by TGOT (Figure 5, bottom trace; n = 2 neurons out of 21; 9.5%), a result which was in good agreement with the small number of c-Fos positive lamina neurons II following PVN stimulation in vivo. It should be noted that although TGOT was able to increase the frequency of occurrence of sEPSPs (spontaneous excitatory postsynaptic potentials) in some recorded neurons, these events did not reach an amplitude or a frequency sufficient to trigger action potentials.

## Discussion

In this study, we show that only a subpopulation of glutamatergic neurons (40–50%) possesses functional presynaptic OT receptors which facilitate the synaptic release of glutamate and ultimately excite a large population of GABAergic interneurons, resulting in generalized increase of synaptic inhibition in lamina II. This broad and massive facilitation of GABAergic inhibition is likely to represent the basis of the documented spinal antinociceptive effect of OT and of PVN stimulation. This effect might include a direct inhibition of superficial dorsal horn interneurons, projection neurons and a selective reduction in Aδ/C fiber-mediated excitation of spinal cord dorsal horn neurons. We also give strong arguments on the neurochemical nature of PVN-activated (OT-sensitive) neurons in the superficial layers of the spinal cord. These OT-activated neurons (seen as c-Fos positive) are mainly localized in lamina I-II and were never GAD positive. This is in good agreement with our electrophysiological result showing that OT excites a specific population of glutamatergic interneurons, tightly interconnected with all GABAergic interneurons in lamina II. This amplification mechanism increasing the level of spinal GABAergic inhibition is likely to explain most of the spinal OT antinociception seen in vivo.

### Presynaptic effect of OT on glutamatergic lamina II neurons

As a first approach, we compared the effect of OT on spontaneous and miniature EPSCs/IPSCs. Whereas miniature currents (i.e. recorded in the presence of TTX) reflect only the activity of individual release sites, spontaneous synaptic currents allow action potentials and network activity-driven neurotransmitter release. TGOT did not change the frequency of occurrence of GABA_A_-R-mediated miniature IPSCs (Figure 2B) but significantly increased AMPA-R-mediated synaptic transmission in 50% of the recorded lamina II neurons (Figure 2A). This suggested the presence of functional OT receptors on the presynaptic terminals of a subpopulation of glutamatergic neurons and the absence of such receptors on the synaptic endings of GABAergic dorsal horn interneurons. Although c-Fos expression was not observed in GAD positive neurons following PVN stimulation (Figure 4), we can not fully exclude that OT receptors are expressed by GABAergic interneurones. Should this be the case, we can however conclude that their activation is certainly not sufficient to directly increase the release probability of GABA (i.e. as seen in miniature transmission experiments; Figure 2) or to induce a significant action potential discharge in these neurons (see Figure 5 and related results in the text). These results are in agreement with those reported previously on cultured laminae I-III dorsal horn neurons [25]. A major difference observed in slices compared to dorsal horn primary cultures concerned the recruitment by OT of a population of AMPA-R mEPSCs with slow rise and decay kinetics (Figure 3). These mEPSCs were rare under control conditions (indicated by the histogram skewness in figure 3A) but were clearly seen after OT receptor stimulation. Their slow kinetics might indicate that they originate at synapses distant from the neuronal cell body, possibly on distal dendrites. In culture, dorsal horn neurons possess simpler dendritic trees and synapses normally impinging on distal dendrites might be established on the cell body and/or proximal dendrites. The existence of silent synapses due to the absence of functional postsynaptic AMPA-R has been previously shown in the spinal cord, in normal and pathological situations [26,27]. Our results indicate for the first time that a pool of "presynaptically silent" synapses, which can be recruited or turned on functionally by a neuromodulator such as OT (Figure 3), might exist in the dorsal horn of the spinal cord. Although we cannot completely exclude a postsynaptic locus for the expression of this phenomenon [28,29], the fast onset and reversibility TGOT effect on glutamatergic transmission argue rather in favor of the unmasking (i.e. by increasing the release probability) of presynaptically silent or whispering synapses at distal dendrites [29].

### A subpopulation of OT-sensitive glutamatergic neurons recruits GABAergic interneurons in lamina II

In contrast with the effect on miniature IPSCs, OT receptor activation induced a massive and generalized increase in spontaneous GABAergic transmission, since it was observed in all lamina II neurons from which we recorded (Figure 2). This result is a key finding our study because it allows us to postulate that OT sensitive neurons, which are unlikely to be GABAergic (i.e. no effect of OT on the frequency of GABAA mIPSCs), are functionally connected (directly or indirectly) to all GABAergic interneurons in lamina II and are responsible for the facilitation of GABA release. Under the same conditions, however, the increase in spontaneous AMPA-R-mediated EPSCs concerned only a subpopulation of neurons (37%) comparable to that in which OT-receptor activation increased the frequency of mEPSCs. Our double immunofluorescence analysis indicates that c-Fos expressing neurons after PVN stimulation did not contain GAD, suggesting that the c-Fos positive neurons were likely to be glutamatergic. An interesting finding of the recent literature is the existence of a class of interneurons displaying no action potential discharge under resting conditions and receiving no apparent input from peripheral sensory fibers even when the latter were stimulated at C fiber strength [24]. These neurons are excited by PVN stimulation and the time-course of their excitation matched that of PVN-induced inhibition of Aδ/C afferent evoked electrical response. As indicated by the results from our current clamp experiments (Figure 5), the neurons directly depolarized by TGOT, represent a very small population of lamina II neurons and this finding is in good agreement with the c-Fos labeling seen in this lamina after PVN stimulation. This result strongly supports the existence of an organized local spinal circuit mediating the inhibitory effects of the descending hypothalamic pathway at the spinal level. Immunohistochemical experiments based on the revelation of c-Fos positive nuclei following PVN stimulation (Figure 4) were in line with our electrophysiological results. Only a small number of neurons was labeled after PVN stimulation and these neurons were located in lamina I and in the outer part of lamina II. It is interesting to note that this area is the main target in the dorsal horn for OT-positive hypothalamo-spinal fibers [8]. Recent work on rat or OT-knock-out mice spinal cord has shown that OT reduced glutamatergic monosynaptic EPSCs evoked by electrical stimulation of primary afferents in the superficial layers of neonatal spinal cord slices [18]. These results are not contradictory to our data since, as discussed in the work of Robinson [18], the presynaptic effect on primary afferent transmission might have been due to the activation of GABAergic neurons exerting an inhibitory effect on synaptic glutamate release from primary afferents.

**Figure 6:**
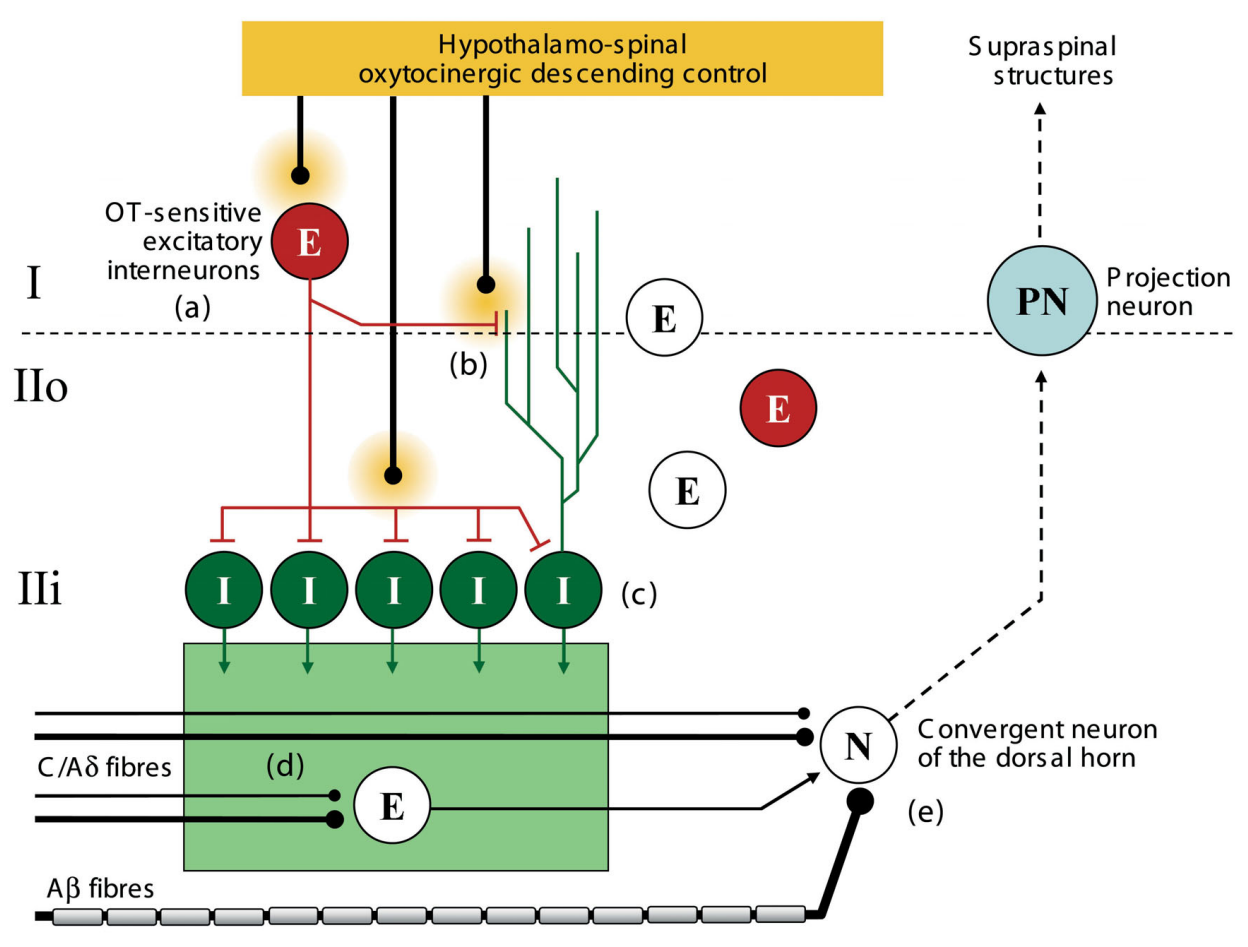
**Simplified diagram proposal summarizing the OT effects seen in lamina I-II at the light of our present study**. Our results indicate that OT, which is physiologically released by hypothalamospinal fibers terminating in lamina I-II, activates a subpopulation of glutamatergic neurons (a) and, in particular, stimulates receptors present on presynaptic glutamatergic synaptic terminals (b). We also show that population of OT-sensitive glutamatergic neurons recruits the whole population of GABAergic neurons (c) resulting in an elevated GABAergic inhibitory tone in lamina II (represented as a green box). The dissection of the spinal action of OT can now unify previous observations which were in apparent contradiction. First, a network driven increase in GABAergic inhibition is likely to explain the specific inhibition of action potential triggered by the nociceptive activation of C and Aδ fibers [21,24]. In this case, a direct presynaptic inhibition of these fibers may be responsible for this effect (d). Alternatively, it remains possible that a specific C/Aδ network is inhibited through presynaptic fibre of neuronal inhibition (d). It remains that neurons, receiving convergent sensory inputs are not the direct target for OT effect since only C/Aδ fibre activity is reduced (e). Second, our findings and mechanism proposal fully answer to the questions left open after the work of Robinson et al [18], i.e. the presynaptic inhibition of glutamate release after stimulation of the dorsal roots and the postsynaptic effect on Lamina I-II neurons (OT sensitive neurons). Abbreviations: E: excitatory glutamatergic neurons; I: inhibitory GABAergic neurons.

Taken together, our results are therefore consistent with the hypothesis that the spinal excitation resulting from peripheral C and Aδ sensory neurons is reduced by a local network involving a subpopulation of glutamatergic neuron activated by OT which excites GABAergic interneurons in lamina II (Figure 6). This synaptic organization might also explain earlier observations made in vivo and showing that application of OT induced either excitation or inhibition of dorsal horn neurons [23].

## Conclusion

In conclusion, we show that a descending hypothalamospinal control using OT as neurotransmitter exerts its effects via a specific local neuronal network in lamina II. This network involves a subpopulation of OT receptor-expressing glutamatergic neurons that distribute their excitation to all GABAergic neurons in lamina II in order to limit or block nociceptive afferent messages originating from C and Aδ primary afferents [21]. These PVN- and OT-activated lamina II interneurons certainly represent an interesting target for the development of specific therapeutic strategies to reduce nociceptive sensory messages at the spinal level.

## Methods

All experiments were conducted in accordance with the recommendations of the International Association for the Study of Pain and directives set by the European Community Council Directive (86/69/EEC of November 24, 1986) and authorization from the French Department of Agriculture (License no. 67–116 to PP).

### Spinal cord slice preparation

In this study we used 15- to 30-day-old Wistar rats anesthetized with a single i.p injection of ketamine (150 mg/kg). A detailed description of the dissection procedure can be found elsewhere [30,31]. Briefly, the spinal cord was removed by hydraulic extrusion and placed into ice-cold sucrose artificial cerebrospinal fluid (s-ACSF) containing (in mM): 248 sucrose, 11 glucose, 23 NaHCO_3_, 2 KCl, 1.25 KH_2_PO_4_, 2 CaCl_2_, and 1.3 MgSO_4 _and was continuously bubbled with 95% O_2_-5% CO_2_. Transverse slices (600 μm thick) were cut from the lumbar segment of the spinal cord using a vibratome and stored at room temperature in an incubation chamber filled with oxygenated (95% O_2_-5% CO_2_) regular ACSF containing (in mM) 126 NaCl, 26 NaHCO_3_, 1.25 NaH_2_PO_4_, 2.5 KCl, 2 CaCl_2_, 2 MgCl_2_, and 10 Glucose. Spinal cord slices were stored for at least 1 hour before being placed into the recording chamber. The slices were continuously superfused with oxygenated ACSF at a flow rate of approximately 3 ml per minute.

### Patch clamp recordings, data acquisition, and analysis

All electrophysiological experiments were performed at room temperature. Lamina II neurons were recorded blindly using the whole-cell configuration of the patch-clamp technique and systematically dialyzed with biocytin in order to localize and determine the morphological characteristics of the recorded interneurons. Synaptic currents were recorded under voltage-clamp using an Axopatch 200B amplifier. The patch pipettes were filled with a solution containing (in mM): 80 Cs_2_SO_4_, 8 CsCl, 2 MgCl_2_, 10 HEPES, 10 Biocytin, and adjusted to pH 7.3 with CsOH. In voltage-clamp mode, lamina II neurons were held at potentials of -60 mV and 0 mV to record glutamatergic and GABAergic synaptic currents, respectively. In a subset of experiments, we have used the current clamp mode to measure changes in the membrane potential (Em). Em was initially adjusted at -60 mV (i.e. with a small current injection if necessary), a value often corresponding to the resting membrane potential in our condition (no current injection: i = 0). For these experiments, cesium was replaced by potassium in the pipette solution described above. Synaptic currents were detected and analyzed individually using the WinEDR and WinWCP softwares (courtesy Dr J. Dempster, University of Strathclyde, Glasgow, UK) to extract their frequency of occurrence, amplitude, monoexponential rise time (τ_R_), and decay time constants (τ_D_). Results are expressed as mean value ± S.E.M. Statistical analysis was performed using parametric one-way ANOVA with Student's *t*-tests to compare means; sets of data were considered as different when p < 0.05.

### Immunocytochemistry following in vivo stimulation of PVN

In anesthetized rats (alpha-chloralose 2% and urethane 20%) a bipolar electrode was implanted in the left paraventricular nucleus to perform 90 minutes stimulation (1 msec, 100 nA, 20 Hz). Immediately after the stimulation, tissues were fixed by an intracardiac perfusion of paraformaldehyde 4%. After a few hours of post fixation in the same fixative, spinal cords were removed, embedded in 5% agar and sectioned with a vibratome in 25 or 30 μm thick sections for direct observations or in 40 μm thick sections for preparation of semithin (1.5 μm) sections. Detailed procedures and antibodies used are indicated in additional file 1.

### Drug application and perfusion solution

To activate oxytocin receptors, we used the selective agonist [Thr^4^, Gly^7^]-oxytocin (TGOT; Bachem, Germany) [32]. OT receptors were selectively inhibited with d(CH_2_)^5^- [Tyr(Me)^2^, Thr^4^-Tyr-NH_2_^9^] ornithine vasotocin [33], a selective OT receptor antagonist (OTA; Sigma). Glutamate-receptor-mediated synaptic transmission was recorded in the continuous presence of the GABA_A_-receptor antagonist bicuculline (BIC: bicuculline methiodide) and of the glycine-receptor antagonist strychnine (strychnine hydrochloride; Sigma). GABAergic synaptic currents were isolated in the presence of the general ionotropic glutamate receptor blocker kynurenic acid (2 mM) and of strychnine (1 μM) to block glycine receptors. To record miniature synaptic transmission, tetrodotoxin (0.5 μM) was present in the bath perfusion at a steady-state.

## Competing interests

The authors declare that they have no competing interests.

## Authors' contributions

JDB, AMV participated in the electrophysiological experiments, PV, SUB designed, carried out and analyzed the immunohistochemical experiments, JDB, PV, RS, MJFM and PP participated in writing of the manuscript. PP conceived the project, supervised the study and drafted the manuscript. All authors read and approved the final manuscript.
